# Exploring the Use of a Guanine-Rich Catalytic DNA for Sulfoxide Preparation

**DOI:** 10.1371/journal.pone.0129695

**Published:** 2015-06-12

**Authors:** María A. Dellafiore, Javier M. Montserrat, Adolfo M. Iribarren

**Affiliations:** 1 Laboratorio de Química de Ácidos Nucleicos, INGEBI (CONICET), Ciudad Autónoma de Buenos Aires, Argentina; 2 Instituto de Ciencias, Universidad Nacional de Gral. Sarmiento, Los Polvorines, Provincia de Buenos Aires, Argentina; 3 Laboratorio de Biotransformaciones, Universidad Nacional de Quilmes, Bernal, Provincia de Buenos Aires, Argentina; University of Pecs Medical School, HUNGARY

## Abstract

A guanine-rich DNA oligonucleotide complexed with hemin was used to catalyze controlled oxygen transfer reactions to different sulfides for sulfoxide preparation in the presence of H_2_O_2_. Comparable activities were obtained when using fully modified L-DNA. In addition, oligonucleotide immobilization led to an active catalyst which could be successfully recovered and reused without loss of activity.

## Introduction

Organic sulfoxides have many interesting applications. They have been used in asymmetrical synthesis for carbon-carbon bond formation, stereoselective transformations [1] and as synthetic intermediates for the production of a wide range of biologically active molecules [2]. They were also found to be effective as anti-ulcer, eugeroic, psychotonic, antimicrobial, hipolipidaemic, anti-atherosclerotic, anti-hypertensive, anti-oxidant, vasodilator, and cardiotonic agents [3–10].

Sulfoxides are mostly prepared by the oxidation of the corresponding sulfides but without a strict control of reaction conditions, such as time, temperature and the relative amount of oxidants, over-oxidation to sulfone generally occurs. Developed techniques tend to have limitations like the use of strong acids, high temperatures, long reaction times, unsafe and contaminant solvents and reagents [11,12].

In the 1990s, Sen and co-workers found a guanine rich oligonucleotide that when complexed with hemin could catalyze peroxidatic reactions to a greater extent than hemin itself [13–15].This DNA molecule, PS2.M, is an 18 mer oligonucleotide that forms an unimolecular G-quadruplex structure composed of three stacked tetrads and four parallel loops, stabilized by potassium. Functioning as an hemin aptamer, the porphyrin stacks upon the terminal guanine tetrad of the quadruplex, to form the DNAzyme [16].

DNAzymes with peroxidase-mimicking activity have great potential in bioanalytical chemistry. They have been widely used for detection of specific DNA sequences, telomerase or methyltransferase activity, determination of metal cations and amplified detection of small molecules in colorimetric and chemiluminescent assays [17].

More recently, another interesting aspect of this DNAzyme as catalyst emerged. First, PS2.M was found to catalyze one-electron oxidations of phenolic substrates with rates superior to horseradish peroxidase [18]. After that, the possibility of the DNAzyme to catalyze oxygen transfer reactions, two-electron oxidations, was investigated. Oxidation of thioanisole, indol and styrene was achieved with kinetics comparable to hemoproteins [19].

Given these results, we intended to further explore the use of this guanine-rich oligonucleotide as a useful catalyst for sulfoxide preparation. Since biologically active molecules are usually bulky, it would be useful to know if sulfides with bigger substituents could be accepted as substrates.

Considering the fact that fully modified PS2.M oligonucleotides are capable of forming G-quadruplex structures and oxidize ABTS [20], it would be interesting to know whether biologically resistant catalysts like these could also perform oxygen transfer reactions.

Finally, the development of an active immobilized DNA enzyme could be useful to take the reaction to a preparative scale.

## Materials and Methods

### Materials

All chemicals and hemin were purchased from Sigma Aldrich unless specified otherwise. D-DNA oligonucleotide was purchased from Integrated DNA technologies and L-DNA oligonucleotide from Chemgenes (DNA Sequence: 5’ GGGTAGGGCGGGTTGGG 3’). Immobilized DNAzyme was synthesized in our laboratory. The support (5’-DMT-deoxyguanosine (*n*-ibu)-3’-non-cleavable long chain spacer on polystyrene) and phosphoramidites were purchased from Chemgenes. Solvents were purchased from J. T. Baker and Sintorgan.

### Methods

All measurements were done at least in duplicate. Error bars are indicated for each individual graphic.

#### Kinetic investigation of D and L-DNAzyme catalyzed oxidation of thioanisole

For D-DNAzyme reaction, a 54 μL volume of a D-DNA stock solution (445 μM aqueous solution) and 24 μL of a hemin stock solution (1mM in DMSO) in 1863 μL oxidation buffer (40 mM HEPES-NH_4_OH, pH 8.0, 20 mM KCl, 0.05% Triton, 1% DMSO)[15] were mixed. For the L-DNAzyme reaction, 104 μL of L-DNA stock solution (230 μM aqueous solution) and 24 μL of hemin stock solution in 1813 μL oxidation buffer were mixed. For “Control Hemin” 24 μL of hemin stock solution and 1917 μL oxidation buffer were mixed and for “Control H_2_O_2_” 1941 μL of oxidation buffer were used. The mixtures were incubated at 25°C for 15 minutes.

To all of them, 50 μL of a thioanisole stock solution (100 mM in DMSO) and 9 μL of 882 mM hydrogen peroxide solution were added to start the reaction. Final reaction volumes were 2 mL and final concentrations were 12 μM DNA, 12 μM hemin, 2.5 mM thioanisole and 4 mM H_2_O_2_. Reactions were carried out in 2 mL glass vials.

Then, 20 μL aliquots were taken at 0, 10, 15, 30, 60, 120, 240, 480, 900 and 1800 seconds and mixed with 19 μL of DMSO and 1 μL of sodium bisulfite 150 mM.

Thioanisole and reaction products were analyzed by RP-HPLC on a Gilson 321 equipped with an UV/VIS-152 detector and an Alltech Apollo C18 column (150x4.6mm 5μm), using a flow of 1 mL/min and the following gradients of a aqueous buffer phase at pH = 5 (A) and acetonitrile (B): 0-1min: 70% A; 1-7min: a phase of A from 70% to 20%; 7–9 min: 20% A; 9-13min: a phase of A from 20% to 70%; 13–15 min: 70% A. For peak detection UV absorption at 254 nm was measured.

Identity of thioanisole (MPS), methyl phenyl sulfoxide (MPSO) and methyl phenyl sulfone (MPSOO) was established using standards, and concentrations were obtained from calibration curves previously performed. Percentages of conversion for each product in relation to the initial concentration of the substrate were calculated. Initial rates (V_obs_) were obtained by calculating the slope of the linear segment of the generation of MPSO vs. time curves.

#### Oxidation of sulfides using D and L-DNAzyme

Stock solutions for diphenyl sulfide (DPS), dibenzyl sulfide (DBS) and dibenzothiophene (DBT) were prepared (100 mM each). Incubations were carried out at 25°C for 15 minutes in the presence of 12 μM Hemin, 12 μM of D or L-DNA oligonucleotide in oxidation buffer using the same stock solutions described for the oxidation of thioanisole. Concentration of substrates was 2.5 mM for MPS, 0.1 mM for DPS, 0.25 mM DBS, and 0.15 mM for DBT. These concentrations were optimized taking into account substrate solubility in the oxidation buffer and the corresponding molar absorption coefficient. Reaction started with the addition of H_2_O_2_ to a final concentration of 4 mM. The final reaction volume was 300 μL. Controls containing the same concentration of hemin and hydrogen peroxide (“Control Hemin”) and peroxide only (“Control H_2_O_2_”) were performed for each sulfide. In these cases, 30 μL aliquots were taken at 0, 1 and 30 minutes and mixed with 1 μL of sodium bisulfite 150 mM.

Sulfides and oxidation products were analyzed under the same conditions as the oxidation of thioanisole. DBS sulfide and DBT oxidations were measured at 220 nm and DPS oxidation was measured at 254 nm.

Percentages of conversion for each product in relation to the initial concentration of the substrate were calculated. In the case of the oxidation of DBT, sulfide and sulfone standards were used to calculate concentrations. For dibenzothiophene sulfoxide (DBTO), standard was not available for purchase. Therefore, the main product peak, with a retention time of 5.57 min, present in the reaction mixture was analyzed using LC-MS giving an m/z = 201.08 which was assigned to [DBTO+H^+^] (S1 Fig), and the percentage of conversion to the sulfoxide was calculated using the mass balance. For the analysis, electrospray ionization with an LCQ Advantage Max Finnigan detector, an MS PUMP Surveryor Plus, a Surveryor Plus autosampler, PDA Surveryor Plus and a Lichrospher 100 RP-15 (5 um) column were used. A flow of 250 μL/min and the following gradients of a water phase with 0.1% formic acid (A) and acetonitrile with 0.1% formic acid (B) were used: 0-1min: 70% A; 1-7min: a phase of A from 70% to 20%; 7–9 min: 20% A; 9-13min: a phase of A from 20% to 70%; 13–15 min: 70% A.

#### Synthesis of immobilized DNA

Synthesis of the DNA oligonucleotide 5’ GGGTAGGGCGGGTTGGG 3’ was performed on Mermade 6 (Bioautomation, USA). DMT-deoxyguanosine (*n*-ibu)-3’-non-cleavable long chain spacer on polystyrene (3 mg) was used as solid support for the immobilized DNAzyme, employing standard phosphotriester oligonucleotide chemistry. Deprotection was carried out in 1 mL of ammonia 30% at 55°C for 8 hours. Dimethoxytrityl cation assay was used to calculate micromoles of full length oligonucleotide present in support resulting in 103 nmoles of immobilized full length DNA.

#### Oxidation of 3,3’,5,5’-tetramethylbenzidine (TMB)

To test the oxidation of TMB in the presence of soluble DNAzymes, 2μL of hemin stock solution, 6.7 μL of D-DNA or 13 μL of L-DNA stock solution in buffer HEPES-NH_4_OH (40 mM HEPES-NH_4_OH, pH 8.0, 20 mM KCl, 0.05% Triton, 1% DMSO) were mixed to a final volume of 40 μL. “Control 1” was prepared with 40 μL of buffer and “Control 2” with 2 μL of hemin stock solution and 38 μL of buffer. They were all incubated 15 minutes at 25°C.

A solution (2 mL) containing 100 μL of TMB stock solution (4 mM in DMSO) and 2.27 μL of hydrogen peroxide solution (882 mM) in citrate-phosphate buffer (0.1 M sodium citrate, 0.2 M NaH_2_PO_4_/Na_2_HPO_4_, 20 mM KCl, pH = 4.4) was prepared. Then, 150 μL of this solution were added to each DNAzyme solution and control to a final volume of 190 μL to start the reaction [21]. Final concentrations were 10.5 μM hemin, 15.75 μM DNA, 0.16 mM TMB and 0.8 mM H_2_O_2_.

Reaction was stopped after 15 minutes by the addition of 100 μL of sulfuric acid 1 M. Absorbance at 450 nm was immediately measured using a Thermo Scientific Nanodrop 1000 UV/VIS spectrophotometer.

For immobilized DNAzyme, 2 μL of hemin stock solution and 38 μL of buffer HEPES-NH_4_OH were added to 103 nmol of D-DNA oligonucleotide immobilized on polystyrene beads. We used the total amount obtained from a solid phase synthesis using 3 mg of support, since it allowed an easier manipulation of the beads. Nevertheless DNAzyme formation was limited by the concentration of hemin, resulting in 10.5 μM as with the soluble enzymes. “Control 3” was prepared with 3 mg of underivatized polystyrene beads in 40 μμL of buffer HEPES-NH_4_OH, “Control 4” was prepared with 3 mg of underivatized beads, 2 μL of hemin stock solution and 38 μL of buffer HEPES-NH_4_OH. “Control 5” was prepared using 103 nmol of immobilized DNAzyme in 40 μL of buffer HEPES-NH_4_OH. They were all incubated 15 minutes at 25°C. Then, 150 μL of the solution containing TMB and hydrogen peroxide were added to each DNAzyme solution and control to a final volume of 190 μL to start the reaction. Final concentrations were 10.5 μM DNAzyme, 0.16 mM TMB and 0.8 mM H_2_O_2_.

This last reaction was done five times to test the possible reuse of the enzyme. Between uses beads were washed with DMSO and then water.

#### Oxidation of thioanisole using immobilized DNAzyme (I-Dz)

Since thioanisole was adsorbed on the beads, 103 nmol of immobilized D-DNA and 1.2 μL of hemin stock solution were incubated with 1 μL of pure thioanisole in oxidation buffer to a final DNAzyme concentration of 12 μM, for 15 minutes at 25°C to saturate the support while allowing DNAzyme formation. Before starting the reaction thioanisole concentration on the supernatant was determined and adjusted to 2.5 mM.

“Control 3” was prepared using 3mg of underivatized polystyrene beads saturated with thioanisole in oxidation buffer with 2.5 mM thioanisole and 4 mM hydrogen peroxide in the supernatant. “Control 4” was prepared the same way and hemin was also added to final concentration of 12 μM. Control 5 was prepared in the same way as the reaction but without adding hemin. After that, hydrogen peroxide was added to every solution to a final concentration of 4 mM. Then, 10 μL aliquots were taken from the supernatant at 0, 1 and 30 minutes and mixed with 19 μL of DMSO and 1 μL of sodium bisulfite 150 mM.

Quantification of thioanisole and oxidation products was achieved in same way as described before.

#### Oxidation of paracetamol using D and L-DNAzyme

Reactions were carried out using 2.5 mM paracetamol, 12 μM hemin, 12 μM oligonucleotide and 4 mM H_2_O_2_ in oxidation buffer. For kinetic measurements, 30 μL aliquots were taken at 0, 1 and 30 and 60 minutes and mixed with 1 μL of sodium bisulfite 150 mM. Each aliquot was analyzed using RP-HPLC with the same equipment previously described and an Alltech Apollo C18 column (150x4.6mm, 5μm) using a flow of 1 mL/min and the following gradients of water pH = 3 (A) and acetonitrile (B): 0–3 min: 5% B; 3-8min: from 5% to 70% B; 8–9 min: 70% B; 9–11 min: from 70% to 5% B; 11–13 min: 5% B. For peak detection UV absorption at 254 nm was used.

Since paracetamol oxidation gives various products, total percentage of conversion was determined integrating the paracetamol peak in each aliquot. “Control Hemin” (without DNA) and “Control H_2_O_2_”(without DNA nor hemin) were performed and treated in the same way as the reactions.

#### Oxidation of paracetamol using immobilized DNAzyme (I-Dz)

Reactions were carried out using 103 nmol of immobilized oligonucleotide, 12 μM hemin, 2.5 mM paracetamol and 4 mM H_2_O_2_ in oxidation buffer. Control for hemin oxidation was prepared using 3mg of underivatized beads instead of I-Dz and control for H_2_O_2_ oxidation was prepared using 3mg of underivatized beads instead of I-Dz in absence of hemin. In all cases, 30 μL aliquots were taken from the liquid phase at 0, 1 and 30 and 60, 120 and 150 minutes and mixed with 1 μL of sodium bisulfite 150 mM. Each aliquot was analyzed using RP-HPLC and percentage conversion was determined.

To assay whether paracetamol was adsorbed on the beads, immobilized oligonucleotide was incubated with the substrate in oxidation buffer. Aliquots were taken at 0 and 24 hours and analyzed using RP-HPLC.

## Results

In this work, we employed a 17 mer sequence variation (5’ GGGTAGGGCGGGTTGGG 3’) [22] of the PS2.M coded as Dz.

### Kinetic investigation of D and L-DNAzyme catalyzed oxidation of thioanisole

To investigate whether L-DNAzyme, a fully modified molecule formed by L-nucleotides, mirror-images of the naturally occurring D-nucleotides, was capable of catalyzing two-electron oxidations, we used thioanisole as substrate. This sulfide is a commonly used test compound for oxygen transfer reactions and was already found to be oxidized by PS2.M [19].

**Fig 1:**
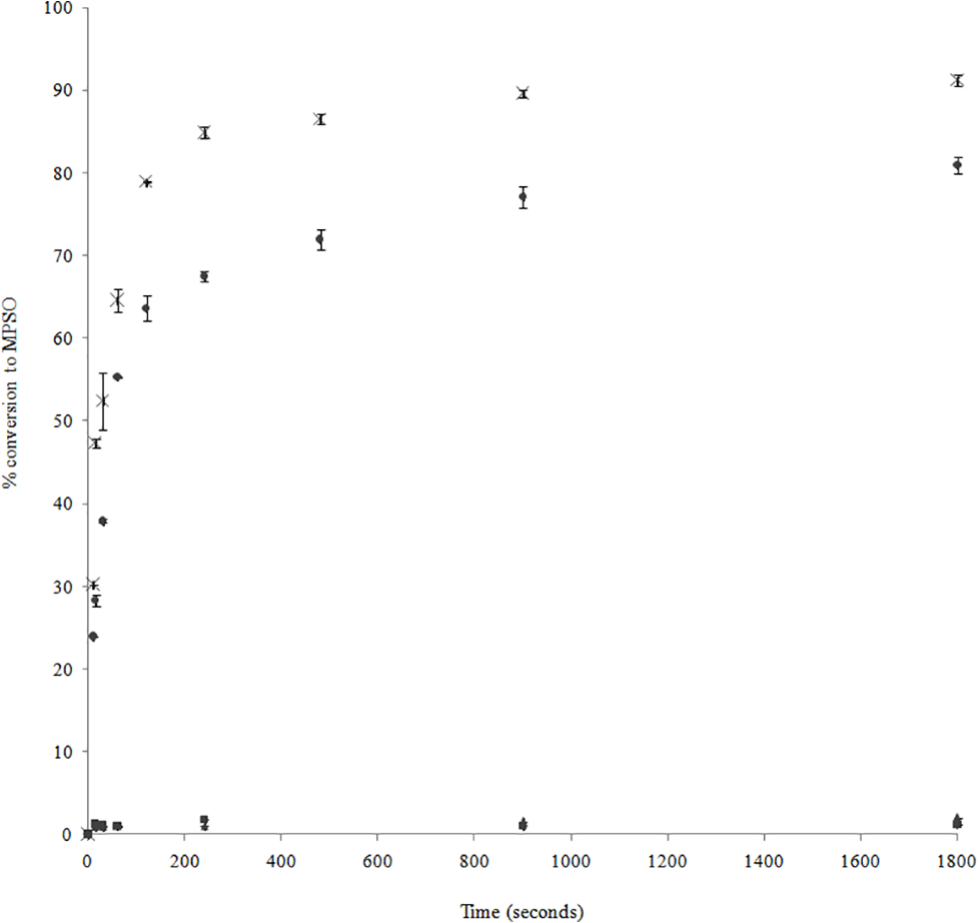
Kinetic study of D and L-DNAzyme catalyzed oxidation of thioanisole. Time courses for the generation of methyl phenyl sulfoxide (MPSO) in the presence of H_2_O_2_ at 25°C catalyzed by D-DNAzyme (x) or L-DNAzyme (●). Controls without DNA, “Control Hemin” (▲), and without DNA nor hemin, “Control H_2_O_2_” (■), were also performed.

First, DNA (12 μM) and hemin (12 μM) were incubated for 15 minutes at 25°C to allow DNAzyme formation. Then, thioanisole was added (2.5 mM) and reaction began with the addition of hydrogen peroxide to a final concentration of 4 mM. Reactions were monitored by RP-HPLC over a 30 minute period revealing rapid appearance of methyl phenyl sulfoxide (MPSO) for both D and L-DNAzyme with comparable rates (Fig 1). Initial rates (Vobs) for D-DNAzyme and L-DNAzyme were 3.1 mM.s^-1^ and 2.0 mM.s^-1^, respectively. Low concentrations of methyl phenyl sulfone (MPSOO) were detected and remained constant throughout the reactions with or without DNAzymes. Conversion to sulfone was circa 12% for the chosen conditions, being independent of DNAzyme or hemin content, and proportional to peroxide concentration (S1 Table).

The reaction mixtures were analyzed to determine if any enantiomeric excess was obtained. In all cases racemic products were obtained, which is in accordance to experiments reported by Poon et al. [19].

### Oxidation of sulfides using D and L-DNAzyme

**Fig 2:**
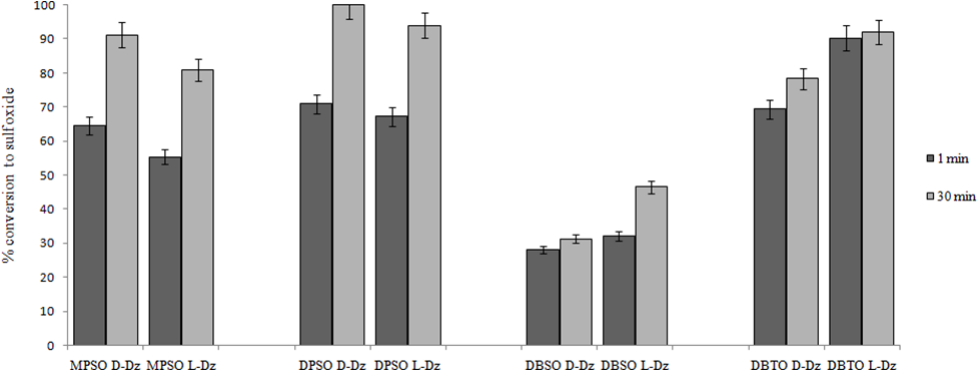
Oxidation of sulfides. Percentage conversion to sulfoxide after 1 and 30 minutes of reaction in the presence of 12 μM of D or L-DNAzyme and 4 mM H_2_O_2_. Concentration of substrates was 2.5 mM for thioanisole (MPS), 0.1 mM for diphenyl sulfide (DPS), 0.25 mM dibenzyl sulfide (DBS), and 0.15 mM for dibenzothiophene (DBT).

Taking into account the previous results, we decided to explore the acceptance of bulkier substrates such as diphenyl sulfide (DPS), dibenzyl sulfide (DBS) and dibenzothiophene (DBT). In all cases, the corresponding sulfoxides were obtained with comparable rates using D or L-DNAzyme (Fig 2).

Percentage of conversion to sulfoxides was always below 3% in controls performed without DNA and without DNA nor hemin. None sulfone formation was observed in the case of diphenyl or dibenzyl sulfides and only low concentrations of dibenzothiophene sulfone were detected (S2 Table).

### Oxidation of 3,3’,5,5’-tetramethylbenzidine (TMB) using immobilized DNAzyme

**Fig 3:**
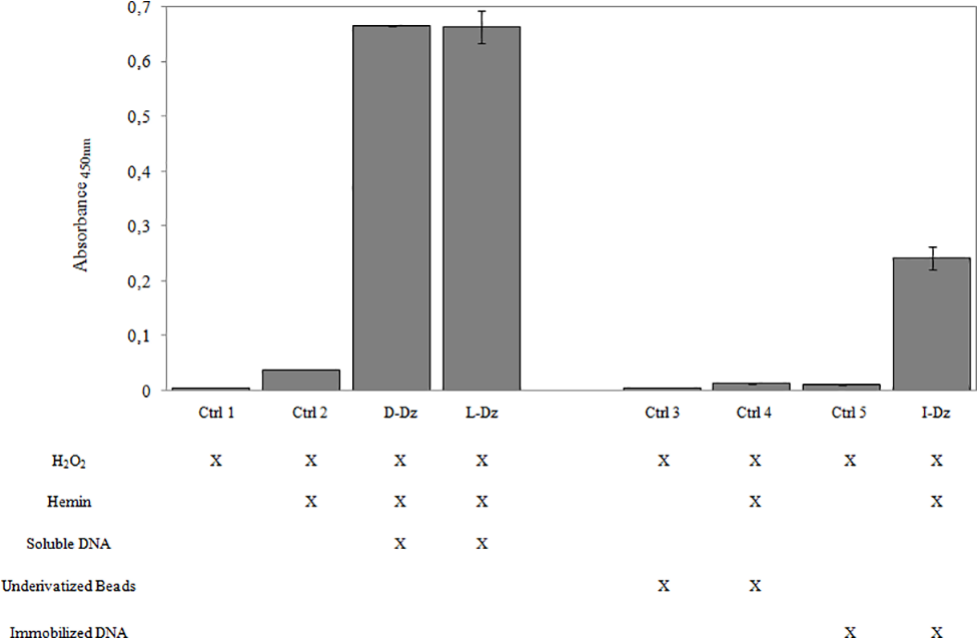
Oxidation of TMB catalyzed by D-DNAzyme (D-Dz), L-DNAzyme (L-Dz) an immobilized DNAzyme (I-Dz). Final concentrations were 10.5 μM DNAzyme, 0.16 mM TMB and 0.8 mM H_2_O_2_. Reactions were stopped after 15 minutes by addition of acid. Absorbance was measured at 450 nm.

Besides its stability, another valuable property of a good catalyst is its possibility of recovery and reuse. Therefore, we were also interested in knowing if this DNAzyme was capable of functioning immobilized on a polystyrene support. For this purpose, the oxidation of 3,3’,5,5’–tetramethylbenzidine (TMB), a widely used colorimetric substrate for peroxidases which allows a rapid and easy detection of oxidation products, was used as a model. In the homogeneous system, after the addition of TMB and hydrogen peroxide, both D and L-DNAzyme containing solutions gradually developed a blue colour indicating the formation of the charge-transfer complex. After incubation for 15 minutes, a strong acid was added and the solutions immediately turned yellow due to the formation of the final product, the diimine (Fig 3). In the case of the immobilized DNAzyme (I-Dz), the beads became blue while the solution remained colorless. This was not observed in controls where beads remained white. After incubation for 15 minutes, acid was added and the liquid phase turned yellow returning the beads to its original white color. In this case the measured absorbance was half compared to the non-immobilized cases (soluble DNA, Fig 3).

To test the possibility of reuse of the catalyst, beads were recovered from the reaction media by centrifugation followed by washes with dimethyl sulfoxide and water before starting a new reaction. After five times no important loss of activity was observed (Table 1).

**Table 1:** Recuperation and reuse of immobilized DNAzyme for the oxidation of TMB.

n° of reuses	Abs _450nm_	Abs.min^-1^
1	0,297	2.0 x 10−2
2	0,256	1.7 x 10−2
3	0,207	1.4 x 10−2
4	0,252	1.7 x 10−2
5	0,199	1.3 x 10−2

Absorbance was measured after 15 minutes of reaction at 450 nm. After each reaction beads were washed with dimethyl sulfoxide and then water.

### Oxidation of thioanisole using immobilized DNAzyme

**Fig 4:**
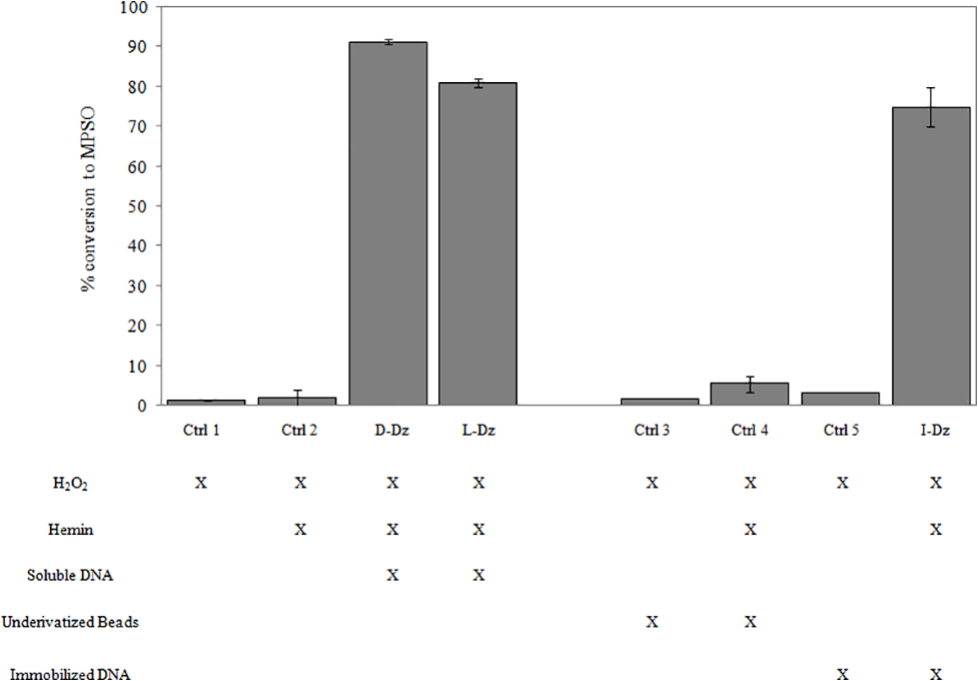
Oxidation of thioanisole using immobilized DNAzyme (I-Dz). Concentration of products was measured after 30 minutes and percentage of conversion was calculated in relation to the initial thioanisole concentration in the liquid phase.

Afterwards, we investigated whether the immobilized DNAzyme was capable of catalyzing oxygen transfer reactions using thioanisole as the test compound. Since the substrate was adsorbed on the beads, they were first saturated and thioanisole concentration in the liquid phase was adjusted to 2.5 mM. The immobilized DNAzyme obtained from one solid phase synthesis (103 nmol) was incubated with hemin (12 μM) and oxidation buffer, giving a final amount equivalent to 12 μM of active DNAzyme. Hydrogen peroxide was added to a final concentration of 4 mM and products were quantified after 30 minutes by RP-HPLC (Fig 4).

This reaction was carried out using the recovered beads from the colorimetric assay and was repeated four times observing no significantly loss of activity (Table 2).

**Table 2:** Recuperation and reuse of immobilized DNAzyme for the oxidation of thioanisole.

	% MPSO	% MPSOO	Activity (%MPSO.min^-1^)
Control 3	1.7	13.8	0.06
Control 4	5.5	8.2	0.18
Control 5	3.2	8.6	0.11
Reuse n°1	88.3	9.8	2.94
Reuse n° 2	66.1	10.0	2.20
Reuse n° 3	69.3	5.4	2.31
Reuse n° 4	75.5	9.2	2.52

After each reaction beads were washed with DMSO and then water. Percentages of methyl phenyl sulfoxide (MPSO) and methyl phenyl sulfone (MPSOO) were calculated in relation of the initial thioanisole concentration in the liquid phase and measured after 30 minutes of reaction.

### Oxidation of paracetamol using D, L and immovilized DNAzyme

**Fig 5:**
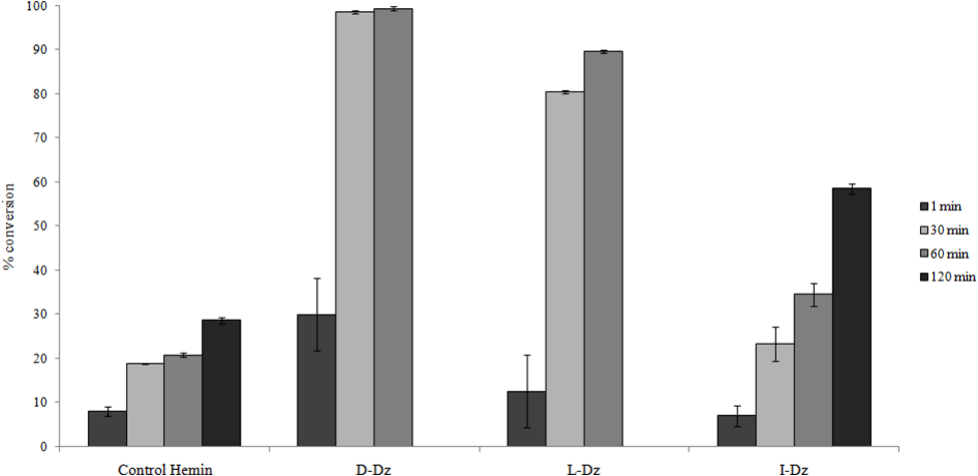
Oxidation of paracetamol using D, L and I-Dz. Percentage conversion to paracetamol oxidation products were measured at 1 min, 30 min and 60 min using D and L-Dz and at 1, 30, 60 and 120 minutes for I-Dz.

It has been recently reported [23] that paracetamol (acetaminophen) is one of the emergent pharmaceutical contaminants in natural water sources. In an attempt to explore the DNAzyme peroxidase oxidative potentiality regarding water decontamination, we have carried out paracetamol degradation using D, L and I-Dz (Fig 5). Although this reaction is different to thioether oxidation, it could be an interesting example of the wide set of oxidations that this DNAzyme can catalyze.

For soluble DNAzymes, reactions were carried out using 12 μM of D or L-DNA, 12 μM hemin, 2.5 mM paracetamol and 4 mM H_2_O_2_ in oxidation buffer. Both reactions reach equilibrium at 60 minutes observing complete oxidation of the substrate in the case of D-Dz and 89.7% conversion for L-Dz. When oxidation was done using hemin without DNA (“Control Hemin”) a percentage conversion of 20.9% at equal time was obtained. Control without DNA nor hemin (“Control H_2_O_2_”) gave no products.

To assay I-Dz activity we used the same immobilized oligonucleotide employed in TMB and tioanisol oxidation. Reactions were carried out in the same conditions as with soluble DNAzymes. In this case the substrate was not adsorbed on the beads. The oxidation reached equilibrium at 120 minutes with a percentage conversion of 58.6. “Control Hemin” and “Control H_2_O_2_” in the presence of underivatized beads gave the same results as before.

## Discussion

Although the L-DNA peroxidase has a lower initial rate than the D-DNA sequence, the final conversion percentages for the oxidation of thioanisole, at times larger than half an hour, are quite similar. For the oxidation of bulkier organic sulfides like diphenyl, dibenzyl and dibenzothiophene sulfides, the corresponding sulfoxides were obtained as the main products, with similar rates when using D or L-DNAzyme.

The activity and reuse of the immobilized DNAzyme peroxidase was initially explored using TMB colorimetric oxidation, obtaining half of the absorbance when compared to soluble DNAzyme (L and D) experiments. These results could be explained taking into account that the blue-colored complex adsorbed on the beads consists of one diimine molecule and one unoxidized TMB molecule. Consequently, lower absorbance values than the ones measured for soluble DNAzymes, were expected after the addition of acid. It is interesting to highlight that blue-colored beads remained constant for at least three months. Therefore, combining this DNAzyme with this particular support, a stable and localized colorimetric reaction was achieved. In all cases, negative controls gave practically no color and the DNAzyme remained active at least after five reuses.

The immobilized DNAzyme was assayed for thioanisole oxidation. The conversion to sulfoxide was measured after 30 minutes finding that the immobilized peroxidase activity was similar to D and L reactions in homogenous phase. When reuse was studied, the DNAzyme was active at least after four reuses, pointing out the stability of the immobilized construction.

Finally, paracetamol degradation using the same catalytic media as thioether oxidation was assayed, finding a complete paracetamol conversion with D-DNAzyme in 60 min while conversion using L-DNAzyme was barely lower. Oxidation was also achieved using immobilized DNAzyme. Although being less active than the soluble ones, its advantage lies on the possible reuse of the catalyst. It should be taken into consideration that the immobilized DNAzyme tested for the TMB, thioanisole and paracetamol oxidation was the same one, emphasizing the reuse stability.

In conclusion, we found that this guanine-rich DNAzyme was capable of catalyzing oxygen transfer reactions to different sulfides, using not only D-DNA but also L-DNA with comparable activity. This result suggests that this approach could also be used in a variety of oligonucleotide degrading media. We achieved thioanisole oxidation using the immobilized oligonucleotide on polystyrene beads, demonstrating the DNAzyme stability over repeated uses and suggesting its potential application in synthetic organic chemistry. In addition, we explored paracetamol oxidation using the same catalytic conditions as in the thioether case.

## Supporting Information

S1 Fig LC-MS analysis of the main product of dibenzothiophene oxidation. (DOCX)

S2 Fig Reaction Schemes. (DOCX)

S1 Table Percentage conversion to methyl phenyl sulfoxide (MPSO) and methyl phenyl sulfone (MPSOO) in the presence of D or L-DNAzyme. Reactions were carried out in the presence of DNA (12 μM), hemin (12 μM), and H_2_O_2_ (4 mM). Controls were performed using hemin (12 μM) with H_2_O_2_ (4 mM) without DNA (Control Hemin), and H_2_O_2_ (4 mM) only (Control H_2_O_2_). Thioanisole concentration was 2.5 mM in all cases.
(DOCX)

S2 Table Percentage conversion to sulfoxides and sulfones after 1 and 30 minutes of reaction in the presence of D or L-DNAzyme. Reactions were carried out in the presence of DNA (12 μM), hemin (12 μM), and H_2_O_2_ (4 mM). Controls were performed using hemin (12 μM) with H_2_O_2_ (4 mM) without DNA (Control Hemin), and H_2_O_2_ (4 mM) only (Control H_2_O_2_). Concentration of substrates, in reactions and controls, was 2.5 mM for thioanisole (MPS), 0.1 mM for diphenyl sulfide (DPS), 0.25 mM dibenzylsulfide (DBS), and 0.15 mM for dibenzothiophene (DBT).
(DOCX)

## References

[1] Marino JP, Viso A, Fernandez de la Pradilla R, Fernandez P. (1991) Asymmetric carbon-carbon bond formation via sulfoxide-directed SN2' displacements of acyclic allylic mesylates. J. Org. Chem. 56: 1349–1351. 10.1021/jo00004a002 11671459

[2] Carreno MC. (1995) Applications of sulfoxides to asymmetric synthesis of biologically active compounds. Chem. Rev. 95: 1717–1760. 10.1021/cr00038a002

[3] Spencer CM, Faulds D. (2000) Esomeprazole. Drugs 60: 321–331. 10983736 10.2165/00003495-200060020-00006

[4] Garnock-Jones KP, Dhillon S, Scott LJ. (2009) Armodafinil. Drugs 23: 793–803. 10.2165/1120329-000000000-00000 19689169

[5] Kiran Kumar ABV, Krishna Rao KSV, Subhosh Chandra M, Subha MCS, Choi YL. (2009) Synthesis and antimicrobial evaluation of sulfides, sulfoxides, and sulfones. J. Korean Soc. Appl. Biol. Chem. 52: 34–39. 10.3839/jksabc.2009.006

[6] Riddell D, Bright CP, Burton BJ, Bush RC, Harris NV, Hele D, et al (1996) Hypolipidaemic properties of a potent and bioavailable alkylsulphinyl-diphenylimidazole ACAT inhibitor (RI 73163) in animals fed diets low in cholesterol. Biochem. Pharmacol. 52: 1177–1186. 8937424 10.1016/0006-2952(96)00455-8

[7] Adetumjbi MA, Lau BH. (1983) Allium sativum (garlic), a natural antibiotic. Med. Hypotheses 12: 222–237. 6366484 10.1016/0306-9877(83)90040-3

[8] Schwartz IF, Hershkovitz R, Iaina A, Gnessin E, Wollman Y, Chernichowski T, et al (2002) Garlic attenuates nitric oxide production in rat cardiac myocytes through inhibition of inducible nitric oxide synthase and the arginine transporter CAT-2 (cationic amino acid transporter-2). Clin. Sci. 102: 487–493. 11980565

[9] Padmanabhan S, Perlman ME, Zhang L, Moore D, Zhou D, Fischer JB, et al (2001) Identification and characterization of a potential ischemia-selective N-methyl-d-aspartate (NMDA) receptor ion-channel blocker, CNS 5788. Bioorg. Med. Chem. Lett. 11: 501–504. 11229757 10.1016/s0960-894x(00)00695-8

[10] Kotelanski B, Grozmann RJ, Cohn JN. (1973) Positive inotropic effect of oral esproquin in normal subjects. Clin. Pharmacol. Ther. 14: 427–433. 4698571 10.1002/cpt1973143427

[11] Kowalski P, Mitka K, Ossowska K, Kolarska Z. (2005) Oxidation of sulfides to sulfoxides. Part 1: Oxidation using halogen derivatives. Tetrahedron 61: 1933–1653. 10.1016/j.tet.2004.11.041

[12] Kaczorowska K, Kolarska Z, Mitka K, Kowalski P. (2005) Oxidation of sulfides to sulfoxides. Part 2: Oxidation by hydrogen peroxide. Tetrahedron 61: 8315–8327. 10.1016/j.tet.2005.05.044

[13] Li Y, Geyer CR, Sen D. (1996) Recognition of anionic porphyrins by DNA aptamers. Biochemistry 35: 6911–6922. 8639643 10.1021/bi960038h

[14] Li Y, Sen D. (1997) Toward an efficient DNAzyme. Biochemistry 36: 5589–5599. 9154943 10.1021/bi962694n

[15] Travascio P, Li Y, Sen D. (1998) DNA-enhanced peroxidase activity of a DNA aptamer-hemin complex. Chem. Biol. 5: 505–517. 9751647 10.1016/s1074-5521(98)90006-0

[16] Sen D, Poon LCH. (2011) RNA and DNA complexes with hemin [Fe(III) heme] are efficient peroxidases and peroxygenases: how do they do it and what does it mean? Crit. Rev. in Biochem. Mol. Biol. 46: 478–492. 10.3109/10409238.2011.618220 21958168

[17] Kosman J, Juskowiak B. (2011) Peroxidase-mimicking DNAzymes for biosensing applications: A review. Anal. Chim. Acta 707: 7–17. 10.1016/j.aca.2011.08.050 22027115

[18] Rojas AM, Gonzalez PA, Antipov E, Klibanov AM. (2007) Specificity of a DNA-based (DNAzyme) peroxidative biocatalyst. Biotechnol. Lett. 29: 227–232. 17091371 10.1007/s10529-006-9228-y

[19] Poon LCH, Methot SP, Morabi-Pazooki W, Pio F, Bennet AJ, Sen D. (2011) Guanine-rich RNAs and DNAs that bind heme robustly catalyze oxygen transfer reactions. J. Am. Chem. Soc. 133: 1877–1884. 10.1021/ja108571a 21265562

[20] Li C, Zhu L, Zhu Z, Fu H, Jenkins G, Wang C, et al (2012) Backbone modification promotes peroxidase activity of G-quadruplex-based DNAzyme. Chem Comm. 48: 8347–8349. 10.1039/c2cc32919k 22792541

[21] Li B, Du Y, Li T, Dong S. (2009) Investigation of 3,3’,5,5’-tetramethylbenzidine as colorimetric substrate for a peroxidatic DNAzyme. Anal. Chim. Acta 651: 234–240. 10.1016/j.aca.2009.09.009 19782817

[22] Zhang M, Li H, Deng M, Weng X, Ma H, Feng S, et al (2012) Studies of the activity of peroxidase-like DNAzyme by modifying 3’- or 5’-end of aptamers. Chem. Biodivers. 9: 170–180. 10.1002/cbdv.201100040 22253114

[23] Taylor D, Serac T. (2014) Human pharmaceutical products in the environment-The “problem” in perspective. Chemosphere 115: 95–99. 10.1016/j.chemosphere.2014.01.011 10.1016/j.chemosphere.2014.01.011 24525259

