# Supplementary material for: Exploring the Use of a Guanine-Rich Catalytic DNA for Sulfoxide Preparation
Source: PLoS One. 2015 Jun 12;10(6):e0129695. doi: 10.1371/journal.pone.0129695 (PMC4466802; doi:10.1371/journal.pone.0129695)
Supplement: S1 Fig — (DOCX) [file pone.0129695.s001.docx]

**S1 Figure. LC-MS analysis of the main product of dibenzothiophene oxidation.** The product peak with a retention time of 5.57 min present in the reaction mix was analyzed using LC-MS giving an m/z=201.08 which was assigned to [DBTO+H^+^].
