# Supplementary material for: Exploring the Use of a Guanine-Rich Catalytic DNA for Sulfoxide Preparation
Source: PLoS One. 2015 Jun 12;10(6):e0129695. doi: 10.1371/journal.pone.0129695 (PMC4466802; doi:10.1371/journal.pone.0129695)
Supplement: S2 Fig — (DOCX) [file pone.0129695.s002.docx]

**S2 Figure. Reaction Schemes**
