# Supplementary material for: Exploring the Use of a Guanine-Rich Catalytic DNA for Sulfoxide Preparation
Source: PLoS One. 2015 Jun 12;10(6):e0129695. doi: 10.1371/journal.pone.0129695 (PMC4466802; doi:10.1371/journal.pone.0129695)
Supplement: S1 Table — Reactions were carried out in the presence of DNA (12 μM), hemin (12 μM), and H2O2 (4 mM). Controls were performed using hemin (12 μM) with H2O2 (4 mM) without DNA (Control Hemin), and H2O2 (4 mM) only (Control H2O2). Thioanisole concentration was 2.5 mM in all cases. (DOCX) [file pone.0129695.s003.docx]

|  | D-Dz | | L-Dz | | Control Hemin | | Control H_2_O_2_ | |
| --- | --- | --- | --- | --- | --- | --- | --- | --- |
| Time (sec) | % MPSO | % MPSOO | % MPSO | % MPSOO | % MPSO | % MPSOO | % MPSO | % MPSOO |
| 0 | 0.0 | 0.0 | 0.0 | 0.0 | 0.0 | 0.0 | 0.0 | 0.0 |
| 10 | 30.3 | 9.0 | 24.0 | 11.4 |  |  |  |  |
| 15 | 47.3 | 10.2 | 28.3 | 12.4 | 0.9 | 11.2 | 1.3 | 12.2 |
| 30 | 52.4 | 10.9 | 37.9 | 13.9 | 0.9 | 10.9 | 1.1 | 12.2 |
| 60 | 64.6 | 10.6 | 55.3 | 12.9 | 1.0 | 11.5 | 1.0 | 11.7 |
| 120 | 78.9 | 10.5 | 63.6 | 11.8 |  |  |  |  |
| 240 | 84.9 | 10.7 | 67.5 | 11.8 | 1.0 | 11.0 | 1.8 | 11.8 |
| 480 | 86.5 | 10.2 | 71.9 | 12.0 |  |  |  |  |
| 900 | 89.6 | 10.0 | 77.1 | 12.2 | 1.5 | 10.5 | 1.1 | 11.8 |
| 1800 | 91.2 | 8.4 | 80.9 | 12.5 | 2.0 | 12.0 | 1.2 | 12.3 |

**S1 Table**. **Percentage conversion to** **methyl phenyl sulfoxide (MPSO) and methyl phenyl sulfone (MPSOO) in the presence of D or L-DNAzyme.** Reactions were carried out in the presence of DNA (12 µM), hemin (12 µM), and H_2_O_2_ (4 mM). Controls were performed using hemin (12 µM) with H_2_O_2_ (4 mM) without DNA (Control Hemin), and H_2_O_2_ (4 mM) only (Control H_2_O_2_). Thioanisole concentration was 2.5 mM in all cases.
