# Supplementary material for: Exploring the Use of a Guanine-Rich Catalytic DNA for Sulfoxide Preparation
Source: PLoS One. 2015 Jun 12;10(6):e0129695. doi: 10.1371/journal.pone.0129695 (PMC4466802; doi:10.1371/journal.pone.0129695)
Supplement: S2 Table — Reactions were carried out in the presence of DNA (12 μM), hemin (12 μM), and H2O2 (4 mM). Controls were performed using hemin (12 μM) with H2O2 (4 mM) without DNA (Control Hemin), and H2O2 (4 mM) only (Control H2O2). Concentration of substrates, in reactions and controls, was 2.5 mM for thioanisole (MPS), 0.1 mM for diphenyl sulfide (DPS), 0.25 mM dibenzylsulfide (DBS), and 0.15 mM for dibenzothiophene (DBT). (DOCX) [file pone.0129695.s004.docx]

|  |  | Thioanisole | | | Diphenil sulfide | | | Dibenzyl sulfide | | | Dibenzothiophene | | |
| --- | --- | --- | --- | --- | --- | --- | --- | --- | --- | --- | --- | --- | --- |
|  | Time (min) | % MPSO | % MPSOO | % Total Product | % DPSO | % DPSOO | % Total Product | % DBSO | % DBSOO | % Total Product | % DBTO | % DBTOO | % Total Product |
| D-Dz | 1 | 64.6 | 10.6 | 75.2 | 70.9 | 0.0 | 70.9 | 28.1 | 0.0 | 28.1 | 69.4 | 8.8 | 78.1 |
|  | 30 | 91.2 | 8.4 | 99.6 | 100.0 | 0.0 | 100.0 | 31.2 | 0.0 | 31.2 | 78.3 | 10.2 | 88.4 |
| L-Dz | 1 | 55.3 | 12.9 | 68.2 | 67.2 | 0.0 | 67.2 | 32.1 | 0.0 | 32.1 | 90.3 | 4.5 | 94.7 |
|  | 30 | 80.9 | 12.5 | 93.4 | 93.9 | 0.0 | 93.9 | 46.5 | 0.0 | 46.5 | 92.0 | 4.5 | 96.5 |
| Control  Hemin | 1 | 1.0 | 11.5 | 12.5 | 1.9 | 0.0 | 1.9 | 0.0 | 0.0 | 0.0 | 1.4 | 0.0 | 1.4 |
|  | 30 | 2.0 | 12.0 | 14.0 | 2.9 | 0.0 | 2.9 | 2.6 | 0.0 | 2.6 | 2.4 | 0.0 | 2.4 |
| Control  H_2_O_2_ | 1 | 1.0 | 11.7 | 12.7 | 0.0 | 0.0 | 0.0 | 0.0 | 0.0 | 0.0 | 0.0 | 0.0 | 0.0 |
|  | 30 | 1.2 | 12.4 | 13.6 | 0.7 | 0.0 | 0.7 | 0.0 | 0.0 | 0.0 | 0.0 | 0.0 | 0.0 |

**S2 Table**. **Percentage conversion to sulfoxides and sulfones after 1 and 30 minutes of reaction in the presence of D or L-DNAzyme.** Reactions were carried out in the presence of DNA (12 µM), hemin (12 µM), and H_2_O_2_ (4 mM). Controls were performed using hemin (12 µM) with H_2_O_2_ (4 mM) without DNA (Control Hemin), and H_2_O_2_ (4 mM) only (Control H_2_O_2_). Concentration of substrates, in reactions and controls, was 2.5 mM for thioanisole (MPS), 0.1 mM for diphenyl sulfide (DPS), 0.25 mM dibenzylsulfide (DBS), and 0.15 mM for dibenzothiophene (DBT).
